# Sexual Risk Behaviors in Adolescent Sexual Minority Males: A Systematic Review and Meta-Analysis

**DOI:** 10.1007/s10935-018-0525-8

**Published:** 2018-11-17

**Authors:** Rachel Valencia, Li Yan Wang, Richard Dunville, Akshay Sharma, Travis Sanchez, Eli Rosenberg

**Affiliations:** 1 Department of Epidemiology, Emory University Rollins School of Public Health, 1518 Clifton Road NE, Atlanta, GA 30322 USA; 2 Division of Adolescent and School Health, Centers for Disease Control and Prevention, Atlanta, GA 30329 USA; 3 Department of Health Behavior and Biological Sciences, University of Michigan School of Nursing, Ann Arbor, MI 48109 USA; 4 Department of Epidemiology and Biostatistics, University at Albany School of Public Health, SUNY, Albany, NY 12144 USA

**Keywords:** HIV risk, United States, MSM (men who have sex with men), Adolescence, Sexual risk behaviors

## Abstract

Although adolescent sexual minority males (ASMM) are at increased risk for human immunodeficiency virus (HIV) in the United States (US), studies that estimate sexual risk behaviors that contribute to HIV risk in ASMM are limited. We completed a systematic review and meta-analysis to compile available data and estimate the prevalence of risk behaviors in this population. We searched four databases for key terms related to ASMM, defined as males aged 14 through 19 who identified as gay or bisexual, reported sex with a male in their lifetime, and/or were considered sexual minority by the study. Articles eligible for inclusion were in English, from US studies, and reported quantitative data on sexual risk behaviors among ASMM. We extracted data from eligible articles and meta-analyzed outcomes reported in three or more articles using random effects. Of 3864 articles identified, 21 were eligible for data extraction. We meta-analyzed nine outcomes. Sixty-two percent of adolescent males self-identifying as gay or bisexual ever had sex with a male, and 67% of participants from ASMM studies recently had sex. Among ASMM who had sex in the last 6 months or were described as sexually active, 44% had condomless anal intercourse in the past 6 months, 50% did not use a condom at last sex, and 32% used alcohol or drugs at their last sexual experience. Available data indicate that sexual risk behaviors are prevalent among ASMM. We need more data to obtain estimates with better precision and generalizability. Understanding HIV risk in ASMM will assist in intervention development and evaluation, and inform behavioral mathematical models.

## Introduction

In the United States (US), the risk of HIV infection in adolescent sexual minority males (ASMM) is high, as evidenced by the few published estimates in this population. While the term ASMM encompasses both identity- and behavioral-based sexual identities, the best available evidence of HIV risk in ASMM is found in incidence and prevalence estimates among young men who have sex with men (MSM), a population defined by behavior. A cohort study conducted in Chicago between 2009 and 2015 reported a baseline HIV prevalence of 7.6% and an incidence rate of 4.1 per 100 person years among MSM aged 16–20 years; the incidence rate among those aged 16 and 17 years was 5.2 per 100 person years (Garofalo, Hotton, Kuhns, Gratzer, & Mustanski, 2016). Using National HIV Surveillance System data, the Centers for Disease Control and Prevention (CDC) estimated that youth aged 13–24 years accounted for 6.7% of people living with HIV in 2009, and 25.7% of the estimated 47,500 new infections in 2010; 72.1% of these infections were attributed to male-to-male sexual contact (Vital signs: HIV infection, testing, and risk behaviors among youths—United States, 2012).

Although few estimates of HIV burden in this population exist, recent research involving MSM aged 18 years and older has shown a high prevalence among the youngest participants, implying substantial risk behaviors in the under-18 subgroup. A study by Oster et al. found an HIV prevalence ranging from 10 to 15% among MSM aged 18–22 years from the Young Men’s Survey and the National HIV Behavioral Surveillance (NHBS) system (2014). Wejnert et al. (2013, 2016) published results from NHBS in 2008, 2011, and 2014, and found an HIV prevalence of 11, 12, and 14%, respectively, among MSM aged 18–24 years. The InvolveMENt study in Atlanta reported an HIV prevalence of 7.4% in Black MSM and 6.3% in White MSM aged 18 and 19 years (Sullivan et al., 2014). The P18 Cohort Study in New York City reported an HIV incidence of 2.9 per 100 person years among MSM aged 18 and 19 years (Halkitis, Kapadia, & Ompad, 2015).

Despite evidence of vulnerability to HIV infection, there is insufficient research evaluating the unique HIV risk and prevention needs of ASMM (Mustanski, Newcomb, Du Bois, Garcia, & Grov, 2011), particularly among those under 16 years. Research in young adult MSM aged 18 or older is of only limited value in understanding younger adolescents, because of the unique circumstances and changes that occur during adolescence: sexual debut, sexual identity formation, continuing cognitive development, and living with parents (Mustanski et al., 2011). However, the regulatory and sampling logistics of conducting research in adolescents younger than 18 are challenging. It can be difficult to obtain approval from some Institutional Review Boards for a waiver of parental permission, which is necessary to enroll youth who are unwilling to disclose their sexual identity to their parents (Fisher & Mustanski, 2014; Mustanski, 2015; Mustanski & Fisher, 2016). It can also be challenging to recruit ASMM, particularly those under 16 years, because many adolescent males might not be willing to reveal their sexual identity or attraction (Mustanski et al., 2011).

In addition to the dearth of academic research among ASMM, there are no existing HIV- or sexually-transmitted-infection-(STI)-specific surveillance systems in the US that collect data on males under 18 years. The Youth Risk Behavior Survey (YRBS) does collect data on a wide range of adolescent health issues, including sexual risk behavior, from population-based samples of high school students across the US (Centers for Disease Control and Prevention, 2013). However, until the 2015 national survey, YRBS did not capture nationally representative data on sexual identity or the sex of sexual contacts.

Estimates of risk parameters are critical to creating and targeting prevention strategies in ASMM. By understanding the drivers of high rates of HIV and other STIs in ASMM, public health officials can target interventions which will yield the highest impact. These estimates are also important for developing models of HIV infection in adolescent populations that can be used to understand the HIV epidemic and interventions for ASMM (Beck, Birkett, Armbruster, & Mustanski, 2015; Jenness et al., 2016). In the absence of widespread, systematically and routinely collected risk data for ASMM, alternative methods must be used to estimate these parameters. To begin to create a comprehensive risk behavior profile for ASMM and to highlight research gaps, we conducted a systematic review identifying existing literature on adolescent sexual behaviors. Specifically, we sought to identify and describe estimates of risk behaviors, catalog the heterogeneity of studies reporting them, and perform a meta-analysis to generate pooled estimates of these behaviors as a starting point in understanding ASMM risk.

## Methods

### Literature Search

We conducted a systematic review to identify articles that reported data on any sexual risk behaviors among ASMM aged 14 through 19 years, including sex with males, condom use, number of sexual partners, age of sexual initiation, and forced sex. For this analysis, the definition of ASMM depended on the selected studies, and included adolescent males who identified as gay or bisexual, reported sex with a male in their lifetime, or were considered sexual minority by the research study. We searched four databases, PubMed, CENTRAL, EMBASE and Web of Science, for relevant literature on March 11, 2015. We did not include grey literature. In each database, we cross referenced comprehensive search terms in six domains: United States, male, male sexual minorities (examples of terms include “homosexuality,” “men who have sex with men,” “gay’), adolescent age (“adolescent,” “young adult,” “teenager,” “high school,” “youth”), sexual behaviors and health outcomes (“sexual behavior,” “sexual partners,” “unsafe sex,” “sexually transmitted disease,” “condoms,” “HIV infections”), and measurement descriptors (“prevalence,” “questionnaire,” “epidemiology,” “data collection,” “cohort studies”). We used medical subject heading (MeSH) terms when searching PubMed and Emtree terms for EMBASE, which are used to index articles in the respective databases. To select these search terms, we initially identified seven relevant articles and tested multiple sets of search terms and logic until all seven articles were present in the output. The systematic review strategy prioritized high sensitivity to capture articles that may report risk behaviors in ASMM even if not mentioned in the title, abstract, or keywords.

### Screening Procedures

The research team screened articles to identify those eligible for data extraction. An article was eligible for extraction if it: was written in English, was about a US study, and reported quantitative data on sexual risk behaviors among ASMM aged 14 through 19 years. There were no additional exclusion criteria. To capture as much literature on this topic as possible, we did not exclude articles based on publication year, study design, sample size, or study quality.

Article exclusion occurred in five stages. First, two reviewers screened article titles for eligibility. Second, two reviewers screened abstracts. Third, for articles that did not have an abstract, two reviewers screened the full text. Fourth, the remaining full-text articles were single reviewed to exclude articles that did not report statistics on individuals 19 years or younger. Last, the remaining full-text articles were dual reviewed for eligibility. In each round with dual review, we excluded articles only if there was agreement between both reviewers to do so. At each stage, reviewers indicated one or more reasons why they excluded an article. If the research team identified relevant articles published after the database search date, we screened them for inclusion.

### Data Extraction

We dual-coded articles that met inclusion criteria into a Microsoft Excel data extraction template. Extracted data included article metadata: authors, year of publication and data collection, source, study design, measurement type, sampling procedures, population description, and definition of ASMM. For each age, race/ethnicity, or sexual identity subpopulation reported, we also extracted sample size, age, race/ethnicity distribution, outcome description, outcome value, measure of variability, and time frame. We compared and reconciled extracted data between two coders and a third party addressed issues when needed.

We extracted any sexual behaviors reported in eligible articles. We reviewed and grouped extracted data by outcome and population. We checked articles for duplicate studies, and when outcomes from a single research study were reported multiple times, only one was selected for inclusion in our meta-analysis.

We considered behavioral outcomes reported in at least three articles for meta-analysis. For each outcome, we harmonized data from individual studies to adjust for differences in time frame, denominator population, and statistics reported. When papers artificially dichotomized or grouped the variables of interest, we converted the frequency data into means and standard deviations, estimating the latter by alternating the low- and high-point of each subgroup (Card, 2012). When articles reported outcomes for ASMM subpopulations separately, such as gay and bisexual adolescents, we combined outcomes and calculated weighted averages and standard errors (Tatebe, 2005). To reconcile different reporting periods for anal sex without a condom, we used an external data source, the InvolveMENt study, to create correction factors. The InvolveMENt cohort study asked the same MSM to describe condomless anal intercourse in multiple reporting intervals; past 30 days, past 3 months, and past 6 months (Sullivan et al., 2014). To contribute as many inputs as possible to our meta-analytic estimate, we applied correction factors to two estimates in our analysis that did not report condomless anal intercourse in the past 6 months. We did not include outcomes that were reported in fewer than three articles in meta-analysis. However, we consolidated and reported relevant risk behavior estimates (e.g., insertive or receptive anal sex) separately in “Appendix.”

### Analysis

We reported study level information (ASMM definition, study design, recruitment source, publication year) and demographics (race/ethnicity, age, location) for each article extracted. When available, we described the distribution of mean age and location of data collection across studies. Among those that reported mean age and sample size, we calculated a weighted average. For race/ethnicity, we estimated the sample sizes of White, Black, Hispanic/Latino, and other using proportions reported in each article, and combined sample sizes across articles to create overall race/ethnicity proportions. For articles reporting YRBS data, we used the unweighted number of responses as sample sizes, while using weighted estimates to calculate meta-analytic estimates. When articles only reported race/ethnicity or age for a larger study group that included ASMM, we assumed the same race/ethnicity and/or age distribution for the ASMM subset.

The research team made a decision to use random effects to calculate pooled estimates a priori because of the variation in sample populations and our goal to make inferences about the wider population (Borenstein, Higgins, & Rothstein, 2009; Card, 2012; Hedges & Vevea, 1998). We calculated combined effect estimates for each outcome in Microsoft Excel using random effects, weighting each study by the inverse of variance, considering within and between study variance (Card, 2012; Hedges & Vevea, 1998). We estimated between-studies variance (τ2) using Hedges and Vevea’s (1998) methods of moment estimator. To calculate combined effect sizes for proportions, we transformed study-specific proportions to logit, and calculated the standard error of the logit using $SE_{l} = \sqrt {{1/N(p) + 1/N({1 - p} )}}$, where *N* is the study sample size and *p* is the reported proportion, and then back-transformed the results (Card, 2012).

## Results

### Systematic Review

**Fig. 1:**
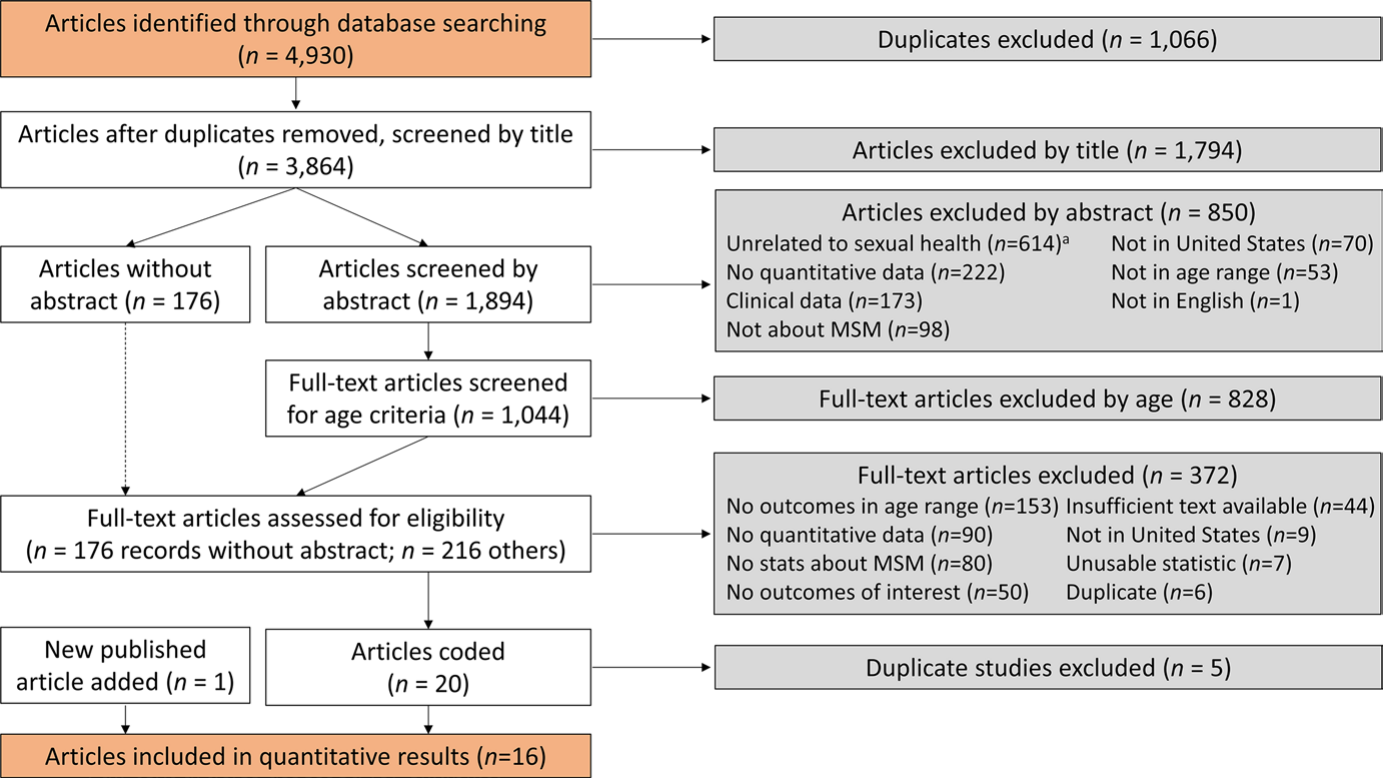
PRISMA flow diagram for systematic review of sexual risk behaviors in adolescent sexual minority males. ^a^Totals do not add up to total articles excluded, since reviewers could select more than one exclusion reason

The PRISMA flow diagram from the systematic literature review is presented in Fig. 1. The initial database search identified 4930 articles. After excluding duplicates (*n *= 1066), we screened 3864 articles for eligibility by title only and both reviewers excluded 1794. We then screened articles with an abstract (*n *= 1894), of which both reviewers excluded 850. The most frequent reasons for excluding articles were because they were unrelated to sexual health (*n *= 614), did not include quantitative data *(n *= 222), and reported only clinical data (*n *= 173). We screened 1044 full-text articles to objectively assess age criteria and excluded 828. We screened the remaining 392 full-text articles, which included 176 without an abstract, and excluded 372 from further data extraction. The most common reasons for full-text article exclusion were lack of reported outcomes for the right age range (*n *= 153), no quantitative sexual behavior data (*n *= 90) and no data on male–male sexual behavior or sexual minority identity (*n *= 80). We included one newly-published article in the meta-analysis. Of the 21 articles coded, 15 contributed to the meta-analytic estimates, and the remaining six articles contributed only to the supplementary outcomes. Of the 21 articles coded, four reported data from the same sample in New York City and three reported from similar pooled YRBS datasets; we used one article from each to calculate demographic and study characteristics. Therefore, we included 16 unique studies in the study metadata and demographic results. Table 1 lists all 21 coded articles.

**Table 1:** Descriptors of articles reporting sexual risk behaviors among adolescent sexual minority males (ASMM)

Article information									
	Author (publication year)	Study type	Recruitment source	Sample population	ASMM definition	Sample size (*n*)	Mean age	Race/ethnicity	City/state/region
ASMM defined by sexual history with another man									
1	Goodenow, Netherland, and Szalacha (2002)	Surveillance-YRBS	School-based	9th to 12th-grade Massachusetts public high school students	Sexual act with male partner	202	16.2	64% White, 10% Black, 7% Hispanic/Latino, 17% other	Massachusetts
2	Halkitis et al. (2011)	Cross-sectional	Community-based, including from providers, street recruitment, participant referral	13–29 YMSM in NYC	Sexual act with male partner	39	No info, all 13–17	5% White, 31% Black, 33% Hispanic/Latino, 31% other	New York City
3	Halkitis et al. (2013)	Baseline data from ongoing prospective cohort study	Community-based, including venue, internet, flyers	18–19 year old males; residents of NYC; self-report of MSM and self-report of negative HIV-serostatus	Report having had sex with another man in the 6-month period before screening	592	No info, but all 18 or 19	29% White, 15% Black, 38% Hispanic/Latino, 18% other	New York City
4	Kann et al. (2016)	Surveillance-YRBS	School-based	Black, white, and Hispanic/Latino male students who had sexual contact with males in grade 9–12	Sexual contact with male in lifetime	1681	No info	14% White, 41% Black, 46% Hispanic/Latino	Multi-city
5	Mustanski, Andrews, Herrick, Stall, and Schnarrs (2014)	Surveillance-YRBS	School-based	Males aged 13 years or older who were sexually active	Sexual contact with male in lifetime	1185	16.1	51% White, 22% Black, 15% Hispanic/Latino, 12% other	Multi-city
6	Pathela and Schillinger (2010)	Surveillance-YRBS	School-based	NYC public high school students	Self-reported sexual act with male	246	15.8	8% White, 32% Black, 47% Hispanic/Latino, 13% other	New York City
7	Sifakis et al. (2007)	Surveilance-YMS	Community-based, through venues	MSM aged 15–29 years in Baltimore, MD	Recruited from venues frequented by MSM; had sex with a man	135	No info, all 15–19	48% White, 39% Black, 3% Hispanic/Latino, 10% other	Baltimore, MD
8	Waldo, McFarland, Katz, MacKellar, and Valleroy (2000)	Cross-sectional	Community-based, through venues	Age 15–22, residents of San Francisco bay area, recruited from venues frequented by young gay and bisexual men	No info; recruited from venues frequented by MSM; had anal intercourse in past 6 months	100	No info, all 15–17	32% White, 23% Black, 31% Hispanic/Latino, 14% other	San Francisco Bay Area
ASMM defined based on gay, bisexual, or other non-heterosexual identity									
9	Arrington-Sanders, Dao et al. (2015)	Cross-sectional survey in a qualitative study	Referral, school-based health clinics, social venues, internet	Black men 15–19 years of age living in Baltimore, MD	Not explicit, but includes self-identified gay, bisexual, other (questioning/intersex)	50	17.6	100% Black	Baltimore, MD
10	Arrington-Sanders, Oidtman et al. (2015)	Cross-sectional survey in a qualitative study	Modified snowball sampling, internet, clinics, venues	Young Black gay and bisexual men age 16–19	Self-identified as gay or bisexual	20	18.4	100% Black	No info
11	Everett, Schnarrs, Rosario, Garofalo, and Mustanski (2014)	Surveillance-YRBS	School-based	12–18 year old male adolescents who reported at least 1 sex partner	Identify as gay or bisexual, or had sexual contact with males in their lifetime; sexually active (at least 1 partner)	855	16.1	49% White, 22% Black, 23% Hispanic/Latino, 6% other	Multi-city
12	Mustanski, Birkett et al. (2014)	Surveillance-YRBS	School-based	13–18 years old adolescents	Self-identify as non-heterosexual (homosexual, bisexual or unsure)	864	16.1	39% White, 19% Black, 27% Hispanic/Latino, 14% other	Multi-city
13	Saewyc, Bearinger et al. (1998)	Cross-sectional	School-based	Public school students in grades 7–12 in randomly selected Minnesota schools, original sample includes lesbian and bisexual females	Self-identified as homosexual or bisexual	212	15.1	79% White, 11% Black, 2% Hispanic/Latino, 7% other	Minnesota
14	Saewyc, Skay, Bearinger, Blum, and Resnick (1998b)	Cross-sectional	School-based	American Indian adolescent males and females who reported ever having had heterosexual intercourse	Self-reported as gay, bisexual or unsure	320	15.1	100% reservation-based American Indian	Indian Health Services areas, multiple locations
15	Thoma and Huebner (2014)	Cross-sectional	Community-based, through venues	Male adolescents reporting as non-heterosexual, 14–19, 4 US cities, living with a parent	Self-identified as non-heterosexual	257	17.4	22% White, 35% Black, 43% other	Multi-city
ASMM defined other									
16	Arrington-Sanders, Trent, Morgan, Harper, and Fortenberry (2014)	Cross-sectional survey in a qualitative study	Modified snowball sampling, internet, clinics, venues	Black gay and bisexual men age 15–19	Not explicit	31	18.1	100% Black	No info
17	Barney (2003)	Cross-sectional	School-based	12–19 year old males who identified as American Indian or Alaska Native	When you think or daydream about sex, think of males only	65	15	100% American Indian and Alaskan Natives	American Indian and Alaska Natives in Minnesota and Alaska
18	Rotheram-Borus, Reid, Rosario, and Kasen (1995)	Cross-sectional	Community-based, LGBT organization	Consecutive series of male youths, aged 14 to 19 seeking services at a LGBT organization	Seeking services at organization for homosexual youths	136	16.8	12% White, 29% Black, 53% Hispanic/Latino, 7% Other	New York City
19	Rotheram-Borus, Reid, and Rosario (1994)	Cross-sectional	Community-based, LGBT organization	Consecutive series of male youths, aged 14 to 19 seeking services at a LGBT organization	Seeking services at organization for homosexual youths	136	16.8	12% White, 31% Black, 51% Hispanic/Latino, 7% other	New York City
20	Rotheram-Borus, Rosario et al. (1994)	Cross-sectional	Community-based, LGBT organization	Consecutive series of male youths, aged 14 to 19 seeking services at a LGBT organization	Seeking services at organization for homosexual youths	131	16.8	12% White, 31% Black, 50% Hispanic/Latino, 7% other	New York City
21	Rotheram-Borus, Rosario, Van Rossem, Reid, and Gillis (1995)	Cross-sectional	Community-based, LGBT organization	Consecutive series of male youths, aged 14 to 19 seeking services at a LGBT organization	Seeking services at organization for homosexual youths	136	16.8	12% White, 30% Black, 51% Hispanic/Latino, 7% other	New York City

### Data Extraction and Outcomes

**Fig. 2:**
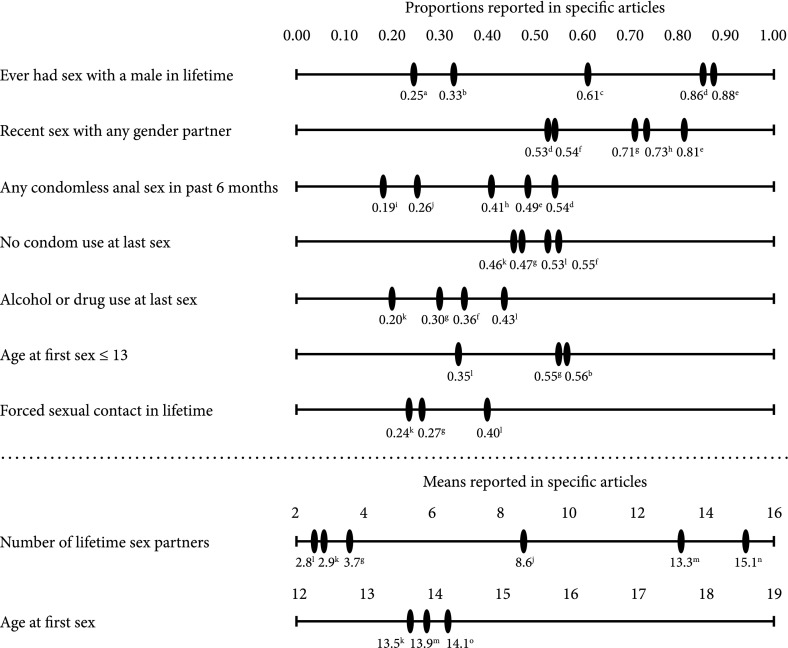
Outcomes identified from articles reporting sexual risk behaviors among adolescent sexual minority males. ^a^Barney (2003), ^b^Saewyc, Bearinger, Heinz, Blum, and Resnick (1998), ^c^Mustanski, Birkett et al. (2014), ^d^Rotheram-Borus, Reid et al. (1995) and Rotheram-Borus, Rosario et al. 1995), ^e^Thoma and Huebner (2014), ^f^Kann et al. (2016), ^g^Pathela and Schillinger (2010), ^h^Sifakis et al. (2007), ^i^Halkitis et al. (2013), ^j^Waldo et al. (2000), ^k^Everett et al. (2014), ^l^Goodenow et al. (2002), ^m^Arrington-Sanders, Dao et al. (2015), ^n^Arrington-Sanders, Oidtman et al. (2015) and Halkitis et al. (2011)

We extracted 57 unique proportion and mean outcomes. Nine of these outcomes had three or more contributing statistics and were eligible for meta-analysis. These outcomes include: ever had sex with a male in lifetime, recent sex, any condomless anal sex in the past 6 months, no condom use at last sex, alcohol or drug use at last sex, age at first sex less than or equal to 13, forced sexual contact in lifetime, mean number of lifetime sexual partners, and mean age of first sex. For each sexual risk behavior outcome, studies reported limited information on denominator populations, partner gender, and type of sex. See Table 2 for detailed definitions of these outcomes. Figure 2 shows the estimates extracted for each of the nine outcomes meta-analyzed. This figure illustrates the consistency of some behavioral estimates reported across different studies (e.g., no condom use at last sex), and the inconsistency of others (e.g., number of lifetime sex partners). “Appendix” includes an additional 48 outcomes that we extracted but that were not eligible for meta-analysis.

**Table 2:** Details of meta-analytic outcomes

Outcome	Details
Ever had sex with a male in lifetime	Ever sex or sexual contact with a male partner was asked in some studies that defined their ASMM study population based on a non-heterosexual identity; this outcome was not reported in studies that defined their ASMM population based on history of sex with a male. How the question was asked, and thus how the outcome is defined, varied by study. Sex or sexual contact with a male partner in lifetime was not defined in three articles, was specified as oral or anal sex in the fourth, and as any genital contact in the fifth
Recent sex	Recent sex was not consistently defined, nor was the population consistent. The recent sex outcome consolidated articles reporting sex in the last 3 or 6 months, and included articles describing recent anal sex, anal sex with males, sex with a male without specifying type, and sex not specifying either gender or type of sex. Recent sex was reported either among people who previously had sex in their lifetime or among all ASMM in the study
Any condomless anal sex in past 6 months	Condomless anal sex was reported among those who had sex in the last 6 or 12 months. One paper specified condomless anal sex with a male while the other four did not specify gender of partner. Three papers mentioned that the studies asked about condomless sex with both receptive and insertive anal sex roles, while two studies did not specify role
No condom use at last sex	Reported among ASMM who had sex in their lifetime, and type of sex and partner gender were not specified
Alcohol or drug use at last sex	Reported among ASMM who had sex in their lifetime, and type of sex and partner gender were not specified
Age at first sex ≤ 13	Articles did not specify type of sex or gender of partner, and articles reported only among ASMM who reported ever having sex
Forced sexual contact in lifetime	Articles did not specify type of sex or gender of person, and articles reported only among ASMM who reported ever having sex
Mean number of lifetime sexual partners	Articles did not specify gender of partners or type of sex
Mean age at first sex	Articles did not consistently report partner gender or type of sex at sexual debut

### Analysis

The 16 unique studies reported in the 21 articles identified were either cross-sectional (*n *= 7), surveillance-based (*n *= 5), or YRBS datasets (*n *= 4). These studies were diverse in how they defined and recruited their study population. Seven studies used an ASMM definition based on history of sexual acts with a male, six were based on identifying as gay or bisexual, one had participants recruited from a lesbian, gay, bisexual and transgender organization, and two had other definitions. Nine studies reported data from studies with community-based recruitment and seven from school-based recruitment. Some research reported on ASMM sub-populations identified through a general population survey, like YRBS administered through schools, while other research studies specifically recruited ASMM from the community and asked questions tailored to this population.

Table 3 describes the demographic characteristics of participants in the 16 unique studies reported in the 21 articles eligible for data extraction. Approximately half of the studies had participants with a mean age of 15 or 16 years old (*n *= 8). Among studies reporting mean age (*n *= 11), the weighted average age was 16.1. Race/ethnicity was distributed widely across articles. Overall, 27% of the combined samples were White, 28% Black, 29% Hispanic/Latino, and 16% other. Three studies had all Black participants; two were comprised of American Indian or Alaska Native participants. Studies were based in multiple locations throughout the country, with four samples recruited in New York City, five with data combined from multiple cities or regions in the US, and two were based in Baltimore.

**Table 3:** Demographic characteristics of articles reporting sexual risk behaviors among adolescent sexual minority males

	Number of studies k	Sample size *n*	Sample distribution %
*Race/ethnicity*			
White	–	1310	27
Black	–	1402	28
Hispanic/latino	–	1443	29
Other	–	783	16
*Mean age*			
15 years old	4	843	17
16 years old	4	1293	26
17 years old	2	307	6
18 years old	3	643	13
No mean reported	3	1855	38
*City/state/region*			
New York City	4	1013	21
Multiple areas	5	3178	64
Baltimore	2	185	4
Minnesota	1	212	4
San Francisco	1	100	2
Massachusetts	1	202	4
No info	2	51	1

Four papers reported on the same sample from New York City, included one in table. Three papers reported on similar pooled YRBS datasets, included one in table

We report nine meta-analytic behavioral risk outcomes estimates in Table 4. The outcomes, and the articles contributing to them, varied by ASMM definition, type of sex, partner gender, time frame, and denominator population. The proportion of ASMM who ever had sex with a male in their lifetime is 62%; we extracted this outcome from studies with identity- or recruitment-based ASMM definitions, whose study respondents may or may not have sexual histories with males. We extracted the remaining outcomes from studies that defined ASMM either based on identity or history of sex with males. Sixty-seven percent of ASMM in the subset of studies reporting this outcome had sex with any gender partner in the last 3 or 6 months, and studies did not consistently specify gender of recent sex partners or report this risk only among ASMM with a history of sex. Forty-four percent of ASMM who had sex in the last 6 or 12 months had any anal sex without a condom in the past six months. Among ASMM who ever had sex, 50% did not use a condom the last time they had sex, and 32% used alcohol or drugs the last time they had sex; the partner gender and type of sex at the most recent sexual encounter was not specified. Forty-nine percent had sex with any gender partner at or before the age of 13, and 30% experienced forced sexual contact by a person of any gender in their lifetime. Among ASMM who ever had sex, the mean age of sexual debut was 13.6 years; type of sex and partner gender was not specified. The mean number of lifetime male and female sexual partners was 6.92. Since this outcome is associated with age of respondents, we calculated the weighted average age among articles contributing to this outcome as 16.14.

**Table 4:** Meta-analytic outcome estimates of sexual risk behaviors in adolescent sexual minority males

Proportion outcomes^a^	Number of studies k	Sample size *n*	Proportion (95% CI)	Tau-Squared
Ever had sex with a male in lifetime	5	1423	0.62 (0.39, 0.81)	1.16
Recent sex with any gender partner (past 3–6 months)	5	2455	0.67 (0.55, 0.78)	0.35
Any condomless anal sex in past 6 months	5	1157	0.44 (0.31, 0.57)	0.33
No condom use at last sex with any gender partner, among those who ever had sex	4	2164	0.50 (0.45, 0.55)	0.03
Alcohol or drug use at last sex with any gender partner, among those who ever had sex	4	2167	0.32 (0.22, 0.43)	0.22
Age at first sex ≤ 13 with any gender partner	3	536	0.49 (0.36, 0.62)	0.19
Forced sexual contact in lifetime with person of any gender	3	1300	0.30 (0.21, 0.41)	0.17

Mean outcomes			Mean (95% CI)	
Number of lifetime sexual partners of any gender, among those who ever had sex	6	1426	6.92 (4.54, 9.30)	7.70
Age at first sex with any gender partner	3	944	13.6 (13.3, 13.8)	0.00

^a^Type of sex not specified for any outcomes listed except for condomless anal sex

## Discussion

While the sexual risk behaviors in ASMM identified in this paper vary by study, the pooled estimates in this meta-analysis are more prevalent than in the general population of adolescent males. An estimated 62% of ASMM in our analysis ever had sex with a male in their lifetime, whereas 42% of males in the 2006–2010 National Survey of Family Growth (NSFG) and 40.9% of heterosexual males in the 2015 YRBS ever had sex with either gender (Kann et al., 2016; Martinez, Copen, & Abma, 2011). An estimated 67% of ASMM in our analysis had recent sex compared to 27.6% of males in NSFG and 30.5% of heterosexual males and 55.3% of males who had sexual contact with only females in YRBS (Kann et al., 2016; Martinez et al., 2011). Only 50% of ASMM used a condom at last sex, compared to 74.7% of adolescent males in NSFG and 62.3% of heterosexual males in YRBS (Kann et al., 2016; Martinez et al., 2011). In addition, 24.2% of heterosexual males in YRBS drank alcohol or used drugs before their last sexual experience, whereas an estimated 32% of ASMM did in our analysis (Kann et al., 2016).

Several sexual risk behavior estimates in this meta-analysis are also higher than those in the 2015 national YRBS, the one nationally representative sample of ASMM sexual risk behaviors (Kann et al., 2016). In YRBS, 47.4% of gay and bisexual males had ever had sexual intercourse, compared to 62% in this analysis. The prevalence of age of sexual debut before 13 was 26.6% among males who had sexual contact with the same sex (Kann et al., 2016), as compared to 49% in this analysis. Recent or current sex was estimated at 67% compared to 58.6% of males who had sexual contact with the same sex (Kann et al., 2016). The different prevalence estimates compared to YRBS may reflect the community-based sampling strategies of some of the studies included in the meta-analysis, which may have recruited higher risk adolescents. Estimates for risk behaviors at last sex were similar in this analysis and YRBS. An estimated 51.4% of ASMM in YRBS used a condom at last sex, compared to 50% in this analysis and 32.2% of males who had sexual contact with a male in YRBS used drug or alcohol compared to 32% in this analysis (Kann et al., 2016).

Approximately half of ASMM had their sexual debut before the age of 13, with an average age of 13.6 years. The more frequent early age of sexual initiation among ASMM may be related to forced sexual experience (Lowry, Dunville, Robin, & Kann, 2016). Age at sexual initiation may be closely tied to nonconsensual encounters, particularly when the age of sexual initiation is under 13 (Finer & Philbin, 2013). The estimated 30% prevalence of forced sexual contact among ASMM is much higher than non-sexual minority adolescent males (3.7% in YRBS) and females (15.8% in YRBS and 20.3% in National Longitudinal Study of Adolescent Health; Kann et al., 2016; Upchurch & Kusunoki, 2004). The estimate is also higher than males who had sexual contact with the same or both sexes in YRBS (16.0%; Kann et al., 2016).

These comparisons indicate that ASMM have markedly higher sexual risk than the general population of adolescent males. Existing research as to the reasons for this population’s elevated risk has been extensively reviewed (Mustanski, 2015). Stigma and discrimination against sexual minority youth play a factor, leading to increased and chronic stress levels (Hatzenbuehler, 2009; Mustanski, 2015). Family support or lack thereof when coming out is a unique experience for ASMM, and has been associated with risky behaviors like unprotected sex and drug use (Mustanski, 2015; Ryan, Huebner, Diaz, & Sanchez, 2009). Lack of ASMM-specific sexual education from schools, medical providers, and family may contribute to limited awareness of HIV transmission risk and prevention strategies (Fuzzell, Fedesco, Alexander, Fortenberry, & Shields, 2016; Kubicek, Beyer, Weiss, Iverson, & Kipke, 2010; Pingel, Thomas, Harmell, & Bauermeister, 2013). Lack of peer support and peer norms for condom use have been associated with condomless anal intercourse and negative sexual health outcomes in MSM (Armstrong, Steiner, Jayne, & Beltran, 2016; Carlos et al., 2010). The prevalence of risk behaviors in ASMM, including recent and condomless sex, alcohol or drug use during sex, and early sexual debut, also place them at a higher risk for HIV infection. Meanwhile, an increasing acceptance and normalization of homosexuality in the US provides an opportunity to reduce sexual risk behaviors among ASMM (Smith, Son, & Kim, 2014). Greater accessibility to gay-identifying teens could allow interventions to better identify youth at risk and target them for age-appropriate prevention strategies.

The findings of this analysis suggest the need to develop effective prevention interventions specifically designed for ASMM. While this research provides prevalence estimates for several key risk behaviors, more precise and generalizable estimates of sexual risk behavior in ASMM are needed. Having strong benchmarks of ASMM risk behaviors can help with research design, intervention development, and serve as baseline estimates of behavior prevalence that future interventions can be evaluated against. Benchmarks also help public health officials target resources for ASMM efficiently. Meta-analytic statistics and other non-pooled statistics identified through the systematic review, and population estimates generated in future research, can help build mathematical models to explore possible intervention strategies, or expand existing models to include adolescent populations. With the increased implementation of pre-exposure prophylaxis (PrEP) as an effective HIV prevention strategy among adult MSM, it is critical to understand the impact it may have on ASMM (Grant et al., 2010). To parameterize mathematical models that quantify the impact of PrEP uptake and other interventions among ASMM on HIV incidence, accurate estimates of ASMM sexual risk behaviors, HIV testing patterns, and sexual networks are needed.

There are several limitations with this analysis. Its primary limitation is that the number of studies included in each meta-analytic estimate is small, so the estimates may not be generalizable and lack precision. Despite this limitation, this research is unique in providing pooled estimates of ASMM risk behavior estimates across research studies. Second, since there is limited research on ASMM available, we did not select articles based on study quality, sampling procedures, ASMM definition, or year of publication. Third, studies used varied definitions of ASMM, defined based on sexual history, identity, place of recruitment, or a combination of these factors. Fourth, the selected studies in this analysis lack consistent and complete measures of sexual risk, and rarely report gender of partners or type of sex with measures like sexual debut. Future studies should, at a minimum, consistently ask partner gender and sex type for sexual behavior measures. Fifth, with the range in publication years, there may be changes in sexual identity expression over time that are not addressed in the analysis. Lastly, data necessary for meta-analysis were unavailable in some of the articles selected. Standard deviations, demographic information for the population subset, sample sizes, and other items were not consistently reported and had to be estimated.

## Conclusion

Based on our review of the literature, this is the first systematic review and meta-analysis of sexual behavior research in ASMM. Consolidating research in this manner highlights the need for more updated, consistent, and in-depth research to truly understand and develop effective interventions that address the increased risk for HIV among ASMM.

## Appendix. Non-pooled sexual risk behavior outcomes in adolescent sexual minority males identified through systematic review

**Table:**

Author (publication year)	Outcome	Population definition	Sample size (*n*)	Proportion, mean (*SD*), median (range)
Arrington-Sanders et al. (2014)	Median partners in past 6 months	Not explicit	31	3 (0–32)
Everett et al. (2014)	More than one sex partner in past 90 days	Identify as gay or bisexual, or had sexual contact with males in their lifetime; sexually active (at least 1 partner)	855	0.212
Goodenow et al. (2002)	Ever been diagnosed with any sexually transmitted disease	Sexual act with male partner	79	0.19
Halkitis et al. (2011)	Mean age of insertive anal sexual debut	Sexual act with male partner	39	15.2 (no *SD*)
	Mean age of receptive anal sexual debut	Sexual act with male partner	39	14.7 (no *SD*)
Halkitis et al. (2013)	Any unprotected insertive anal sex in past 30 days	Report having had sex with another man in the 6-month period before screening	592	0.11
	Any unprotected receptive anal sex in past 30 days	Report having had sex with another man in the 6-month period before screening	592	0.14
	Any unprotected receptive AND insertive anal sex in past 30 days	Report having had sex with another man in the 6-month period before screening	592	0.05
	Mean age of insertive anal sexual debut	Report having had sex with another man in the 6-month period before screening	592	16.3 (1.7)
	Mean age of receptive anal sexual debut	Report having had sex with another man in the 6-month period before screening	592	16.2 (1.8)
Kann et al. (2016)	Had sexual intercourse with four or more persons during their life	Sexual contact with male in lifetime	1681	0.35
Mustanski, Andrews et al. (2014a)	Risky sex (defined as no condom use at last sexual event and 2 + sexual partners in past 3 months)	Sex with male in lifetime	1185	0.16
	Intimate partner violence in past 12 months	Sex with male in lifetime	1185	0.25
Rotheram-Borus, Reid et al. (1994)	Consistent condom use during anal sex (condoms used during all anal sex acts) in past 3 months	Self-identified as gay or bisexual and had anal sex in past 3 months	71	0.45
	Proportion of anal sex acts that were protected in past 3 months	Self-identified as gay or bisexual and had anal sex in past 3 months	71	0.59
	Mean # of male sex partners (anal or oral) in past 3 months	Self-identified as gay or bisexual	135	2.4 (no *SD*)
	Mean # of anal or oral sex acts in past 3 months	Self-identified as gay or bisexual	72	3.5 (5.5)
	Mean # of unprotected anal sex acts in past 3 months	Self-identified as gay or bisexual	68	3.5 (no *SD*)
Rotheram-Borus, Rosario et al. (1994)	Any condom use by partner in lifetime	Self-identified as non-heterosexual, and who engaged in sexual activity where condom could be used by partner	115	0.84
	Any condom use by partner in past 3 months	Self-identified as non-heterosexual, and who engaged in sexual activity where condom could be used by partner	86	0.64
	Any condom use by self in lifetime	Self-identified as non-heterosexual, and who engaged in sexual activity where condom could be used by self	110	0.87
	Any condom use by self in past 3 months	Self-identified as non-heterosexual, and who engaged in sexual activity where condom could be used by self	80	0.51
	Any insertive anal sex in lifetime	Self-identified as non-heterosexual	131	0.66
	Any insertive anal sex in past 3 months	Self-identified as non-heterosexual	131	0.34
	Any receptive anal sex in lifetime	Self-identified as non-heterosexual	131	0.73
	Any receptive anal sex in past 3 months	Self-identified as non-heterosexual	131	0.44
	Reported only one male partner in past 3 months	Self-identified as non-heterosexual and had sex in past 3 months	91	0.37
	Reported 5 or more male partners in past 3 months	Self-identified as non-heterosexual and had sex in past 3 months	91	0.27
	Reported 4 + male receptive anal sex partners in past 3 months	Self-identified as non-heterosexual and had receptive anal sex in past 3 months	57	0.19
	Reported 4 + male insertive anal sex partners in past 3 months	Self-identified as non-heterosexual and had insertive anal sex in past 3 months	91	0.09
	Median lifetime male partners	Self-identified as non-heterosexual; sexually active	117	7 (1–3000)
	Mean lifetime male partners	Self-identified as non-heterosexual; sexually active	117	70.1 (340.9)
	Median male partners past 3 months	Self-identified as non-heterosexual; sexually active with males	91	2 (1–631)
	Mean male partners past 3 months	Self-identified as non-heterosexual; sexually active with males	91	10.3 (65.9)
	Median sexual encounters with males in lifetime	Self-identified as non-heterosexual; sexually active with males	114	39 (1–3120)
	Median sexual encounters with males in past 3 months	Self-identified as non-heterosexual; sexually active with males in past 3 months	91	8 (no range reported)
Rotheram-Borus, Rosario et al. (1995)	Mean # of male sex acts/encounters in past 3 months	Self identified as homosexual or bisexual	136	7.2 (10)
	Mean # of male sex partners in past 3 months	Self identified as homosexual or bisexual	136	1.9 (2.3)
	Mean # of unprotected anal sex acts in past 3 months	Self identified as homosexual or bisexual	136	1.4 (3.3)
	Mean # of male unprotected sex acts in past 3 months	Self identified as homosexual or bisexual	136	7.6 (11.8)
	Mean # of unprotected oral sex acts in past 3 months	Self identified as homosexual or bisexual	136	5.6 (8.3)
Saewyc, Skay et al. (1998)	History of sexual abuse	Self-report as gay, bisexual or unsure	320	0.08
	Weekly intercourse	Self-report as gay, bisexual or unsure	320	0.12
Waldo et al. (2000)	Any unprotected insertive anal sex in past 6 months	Self-identified as gay or bisexual	100	0.20
	Any unprotected receptive anal sex in past 6 months	Self-identified as gay or bisexual	100	0.22
	Ever had a STI	Self-identified as gay or bisexual	100	0.05
	Known HIV-positive sex partner in past 6 months	Self-identified as gay or bisexual	100	0.02
	Mean # of male sex partners in past 6 months	Self-identified as gay or bisexual	100	2.4 (4.2)
